# Supplementary material for: Alterations in protein N-glycosylation confer vanadate resistance in Ogataea polymorpha mutants defective in phosphomannosylation
Source: Front Mol Biosci. 2026 Jan 16;13:1741711. doi: 10.3389/fmolb.2026.1741711 (PMC12855064; doi:10.3389/fmolb.2026.1741711)
Supplement: Supplementary file 1 [file DataSheet1.pdf]

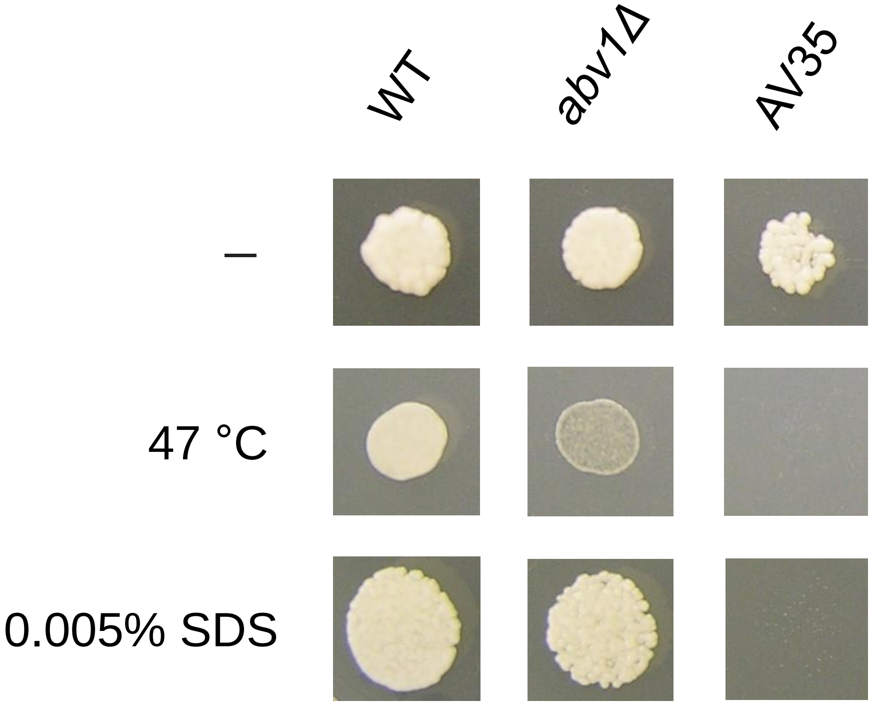

Figure S1. Sensitivity of the AV35 mutant to elevated temperature and presence of SDS in culture medium. Overnight YPD cultures of the AV35 mutant, as well of the 1B27 (WT) and 1B27-620M1 (*abv1Δ*), were 100-fold diluted and 2 μL of the obtained cell suspensions were applied onto the surface of 3 plates, two of which contained YPD without supplements, while the third contained YPD supplemented with 0.005% SDS. The SDS-containing plate and the one of the plates with plain YPD (–) were incubated for 2 days at 37°C; another plate with plain YPD was incubated for 2 days at 47°C.

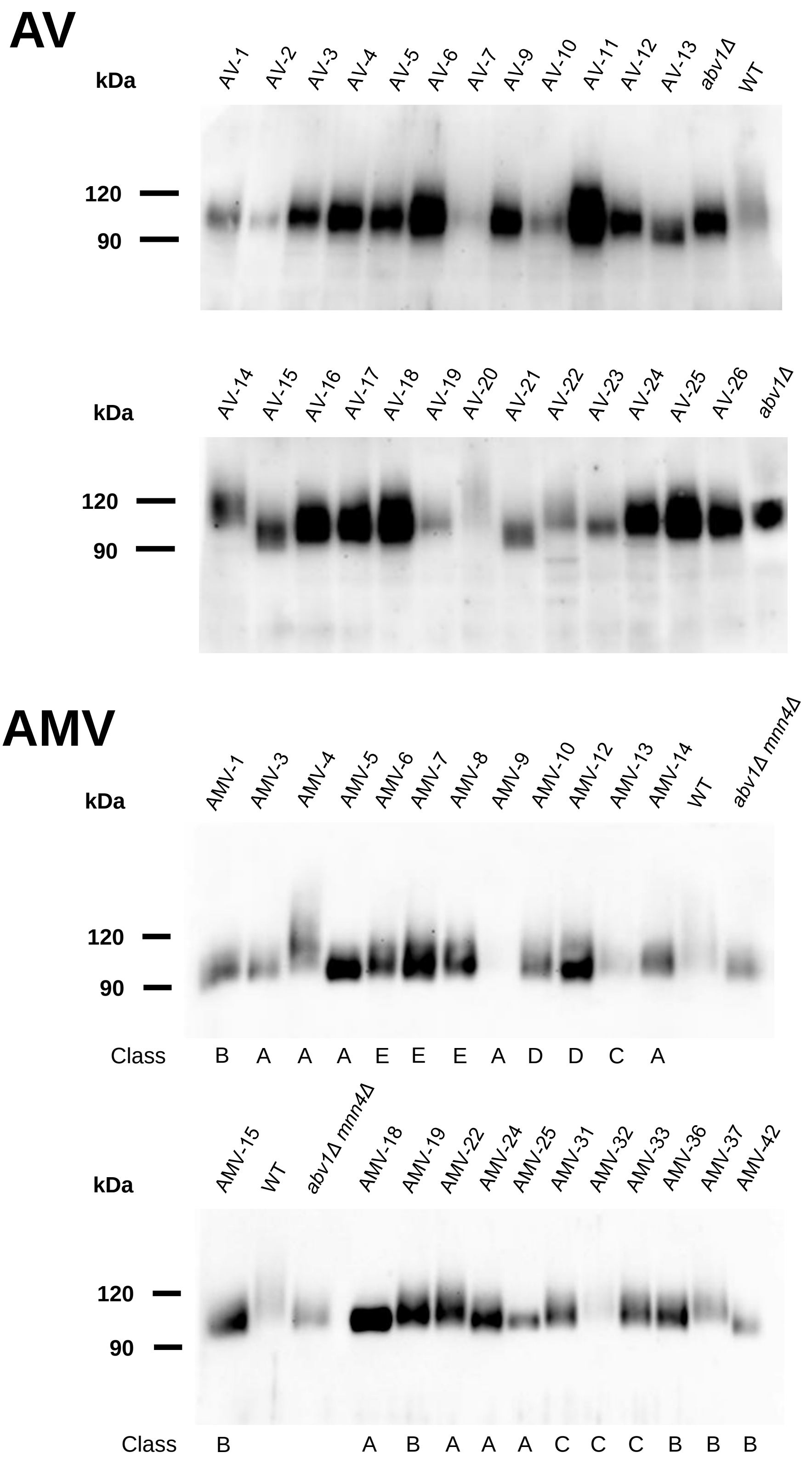

Figure S2. SDS-PAGE and immunoblotting of extracellular GOX. AV, culture supernatants of mutants obtained in the *abv1Δ* mutant M257-620M1, whose culture medium was used as a control (*abv1Δ*). AMV, culture supernatants of mutants obtained in the *abv1Δ mnn4Δ* mutant M257-620-MP6, whose culture medium was used as a control (*abv1Δ mnn4Δ*). Culture medium of the M257 strain with wild-type glycosylation (WT) was used for comparison. Classes of the AMV mutants are indicated at the bottom: class A, clones exhibiting sensitivity to both elevated temperature and SDS; B, clones sensitive to SDS only; C, clones sensitive to to elevated temperature only; D, clones growing in these stress conditions similar to the original strain; E, growing better that the original strain.

A

Clone 5 R
gatcgtcttggtctcgctgttttgccgcaatttttttatttcctcgtgtatatccaaggattctagcatatcttgttcatcttgaagcccaagagtgcgcaacct
cactcgtctggtggttctcttggctgttttcttacgaatgataacgtcttcttgtttgatttcgtcctctgggagctcctcatgaacagcctccacaatctcctc
tccatcctgactttttgtctctgcaactccttaatctcgttgttcagctcatacagtgtacgcctgcctgcccggcgaatgatcggactgggctcgacaacttt
actcggagacacatccatcacttgaggactctttttctcaggcgtcaggttgccgaaaaacattgcgctcgatgtatttgattctgttcgtaggtattgtggagt
ttcctgcaaaagcgcggttctctcacgacggggtgtctcctgaaatagaagcttgcgttcaactttttcgtgggagtct

Clone 5 L
gatccgtatttcccttttgggtatcagaccaaccgaataagaaggccctgtggctgatgctgttgtggctagataatagagaaggaaaaaaaagtgcaatttcaa
tacattttaacttaaaaaaacactccatcaactggtggtgcgctgattctcccacagcctacgtgtgtctgctatatcaatcactagttttaagatttttttgga
cttgtacttctattctgcacagacgacaggctgggaagttttctgcgacgactacgcaaaaggcataacaagggaaaataaacaatccaaaccaggcagcagctc
gcaagatattgttgatgtctatgttgtttgcaagaaacagaagagccaagacgttgatcttggtggtgtcgattctgatcacggtactggtcctttcttcgtccg
ccggtacagaacacctgtcgaaatttaaactgcctacttccagctcctactcgatatatgcagcctccgaagtgaaagag

Clone 6 R
gatcttcttgtatttgtgatatttttttatatcttgccgagacggctaagtcagccttgatcacatcggtagtgggcattttaccgttctttgcttcatacgcatg
ctcccagtgctttatttcgcgcttcaaggagctcacataccgctccaatttgttgatctccatcgaaacgcgtcaaaatagtgtgaataaaattgatagatggag
ttacgcgcagatcacatgacaagcgcgaatattgaaaatcgtttaaattctttgtagggcagttactagcatagagacactgagcggatgggcttgcgccatata
ggcatatcccgaagccagcaatacgccatttgccgataaatgcatgtcttacagattaaatattattattatattacccagaaaagtctatctgttgttactcaa
cagcaacatcatccggtgtctcaaccttttcagccttctctggagattcaacaaggtcgggtggaggagacttgttgtga

Clone 6 L
gatcttgtagatcaaggactctctatctgtggggacgcccccgctaatggcttccagtactatgacaagtagcaggagcacgagaagtcctaaacgcaccagtct
tcttggaatatgaagcatacgagacaaaattgtacgtagcactgtctgatttctataatctggccaggaagcccagaacgggtcttgcttgagctgtgtttaatt
ggctattgtgaccaacaataagatgtgcatagaataaaaaaaaaaaatagcagccactccgcatacggaagaatggtctgtttgcaaaagtttgaatcaagaaac
agtgcgtttggataccgccggcatcaatagcagttcggatagagcgtcggtggctaaataacatgcaggcaggatatctgcaattcgtacacctctacaaattaa
ttacctcgtatcagagtctataaaagtctgcttatgtgacgctgtcaggtggactgaattttctgttttctattgctcca

B

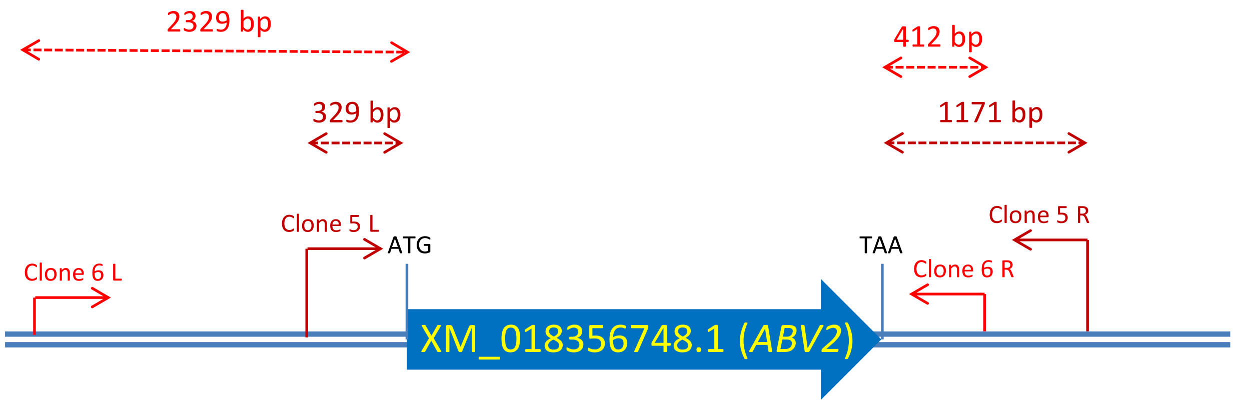

Figure S3. Sequences of the insert flanks in the plasmids recovered from *O. polymorpha* genomic library by complementation of the AV35 mutant phenotypes (A) and their position respectively the XM_018356748.1 open reading frame (B).

```
                1        10        20        30
OpoAbv2    .....MSMLFA....RNRRAKTLILVVSILITVLVLSSSAGTEHL...............
OpoMnn2A   .....MGSYVRLL.RRRGVFRALVAVVLTLVSWLLLPRRS...............RRGL.
OpoMnn2B   MWITKLNLKYRT...KQRVRISLILACAAFMLLFLVVRSFFLENDE.....RVAGSSAIV
ScMnn2     .......MLLTK...RFSKLFKLTFIVLILCGLFVITNKYMDEN................
ScMnn5     .......MLIRL...KKRKILQVIVSAVVLILFFCSVHNDVSSSWL..............
CaMnn2     MI.............AKQKIKILIGVIIVIATYHFIVSSNV..........RSKDLSDLV
CaMnn21    MF.QQLTYRLRLFRRRHKYIFINSIFLSVIIIFLIYSYWSNL.........PAEDNSAII
CaMnn22    .....MGSIFKD...GRRILVRPKSLIICLCLISIIFTQLIR..................
CaMnn23    .....MSINFLSI.PRNRFKAIGVLSVTCILIYVILHSSII...................
CaMnn24    M....FSIPVSS..KTVRLILVSLLLITLINILAAFQRSTLSSWFP.........SSRHI
CaMnn26    .......MSLRRL.SPSHLILGTLVLGVIIFNLYVLTSTHEDIKKVKGPTYHTSDNTKIQ

                 40                50                   60        70
OpoAbv2    ....SKFKLPTSSS........YSIYAASEVK...........EEPSGSAAPSSTPEDAE
OpoMnn2A   ....SYYEQHLRSSSL......SRPFSGLRAG...........EAGLDEL..LEYEHSLS
OpoMnn2B   V...................................................NNDLAQR.
ScMnn2     ......................TSVKEYKEYL...........DRYVQSY..SNKYSSSS
ScMnn5     ..............................YG...........KKLRLPVLTRSNLKN..
CaMnn2     .DLGSSDKSTTEN..............................ERPKNNIVTNNRLDNPP
CaMnn21    NEKGTYHRSLWESITMALFPPKTKPFEEKKPQVNPNNNQEVGVESGASEI..SQHKQQQQ
CaMnn22    .........................YQYQLIA...........DEVQPTI..NEDHSSSQ
CaMnn23    ......................TTDFDVSDYG...........DKFIPSIIFDDNNDNGE
CaMnn24    INKFTDLRLALSSQESVLRDEEGEIYSLVGYHHDFDSNLVVVQKQYLLEK..TPNEDTTE
CaMnn26    SH....................ISNYDSEEYV...........DRLTAEIEDAKKEELIS

                80        90       100
OpoAbv2    NLRNLYFSPEQGGVIPGGSSGNTKGWKVSSIDESK.........................
OpoMnn2A   ELRNSQGL....................................................
OpoMnn2B   .................PTSKKTAKELLAQKKKTR.........................
ScMnn2     ............................................................
ScMnn5     ............................................................
CaMnn2     NEDIPHA.....................................................
CaMnn21    QQQHAKE..........PTTKTSSKSLVSD..............................
CaMnn22    SLKNTK......................................................
CaMnn23    NLKDP.......................................................
CaMnn24    HFWNFLQ..........SNFETKSEYDLNLIDG...........................
CaMnn26    EIRKKLEIQEKPGVIQ....KLKTELRMKYIDDIKNHLKQEITEQYSNEIFKQYAFSFEI

                                    110       120       130       140
OpoAbv2    ..........................GQRTDDRSIPLTENQSPTEEALNKITPEDIVRGD
OpoMnn2A   ......................................QPA....RYVGVAKTDT.....
OpoMnn2B   ..........................ESVYDNLSVEDREKM....RVINQPSPDDPRGLR
ScMnn2     ..........................DAASADDSTPLRDND................EAG
ScMnn5     ............................................................
CaMnn2     ......................................EPD........SPPQEPPKSGN
CaMnn21    ..........................EAYQLNLKLINEQQE.......KYNRLHPFEQKR
CaMnn22    ...........................................LNSTRSSSPISPPKSLN
CaMnn23    ......................................QPE..LDNDKGNGETDTTTSNS
CaMnn24    ..........................YNYKKLIKHLNEQNE..LQLSHSFVEQYKMENQF
CaMnn26    YSKKVDEYALQLENSLKPASCLAILQQAEKDTDSIPDLENY.L...........TKANDK

                 150        160                       170
OpoAbv2    V.TFQNFFIEIF.ELMRKNQLS..............YPLAE..RQTLKDGKP........
OpoMnn2A   ..QFQKYFKDIW.ATILANKPRTGFELTPGVAVGSGMPLEVLVKREYGAYKA........
OpoMnn2B   LELGHTFFKSVF.NVLDKGKPRVS.............PLT.....KYKSTER........
ScMnn2     NEKLKSFYNNVF.NFLMVDSPKGS..................TAKQYNEACLL.......
ScMnn5     .....NFYTTLV.QAIVENKPADS............SPDLS..KLHGAEGCS........
CaMnn2     KPDFSIFFEGL..EKFAIKQPGIK....................DKYTSEKA........
CaMnn21    LQQGYEFFDNIF.KIFYQAKPSVS.............QLN.....TYPSKKR........
CaMnn22    KLTSQEFWEHIF.NIFEINKFDDGLK...........PLI.....KYTPKEQ.QL.....
CaMnn23    MTTAHTFWKGIF.DTFDKYKMDLGQD...........PEN...AVSYVDKLK.QK.....
CaMnn24    IQSFQNFFVQLI.DTIEDCKPDLD.............PINN..DNHYPNGDKIVKYYELR
CaMnn26    YFKRQEYWRYLLKDILLNNKPKCE.............PLT.....KEEKGEK........

                                   180        190         200       210
OpoAbv2    .......................FIENVLFFAQP.WDRL.SEEDCL.QFVNFPPQFINDL
OpoMnn2A   .........................VNVAMHDTVGVPLL.SEEYLL.NCLKVPPEMVADL
OpoMnn2B   ........................IYHAGYEVMGDGPSL.SKEYLS.RFLQLSRSEINSM
ScMnn2     ......................KGDIGDRPDHYKDLYKL.SAKELS.KCLELSPDEVASL
ScMnn5     .......................FANNVAAHDSGHDSDL.SYESLS.KCYNLNKTVQESL
CaMnn2     ............................KEKFSTDDNFLFGKEYLE.NVLDIPQATFKEL
CaMnn21    ........................IYHARFDSLADDDTIFSEKYLS.QFLQLSNEELAAM
CaMnn22    ...................................TTKIKTRDWLL.SKANIFHKDI..I
CaMnn23    ...................................QGPL.NKEVLL.SKAIVSSELMKHL
CaMnn24    NKIPSENMKQFNIERLIHRNGRIPIYGGHLREQYKDELIRNKEFLS.MYLTLSDSEISAL
CaMnn26    ...........................LNPTYQWDARII.SEQYLLGSKLTIPGEKFRAL
```

```
                  220                        230                     240
OpoAbv2    TLKHETVV.SNLPK..........ITP.....RFYKGN.........GYVIVGGGKYSWF
OpoMnn2A   AVSHRNVV.RDLPA..........SYP....EGLYEGR.........GIVFVGGGKFSWL
OpoMnn2B   KKSHSFVT.RNLPE..........TYP....IGLYSGN.........GVVYVGGGKFNWL
ScMnn2     TKSHKDYV.EHIATL.........VSP....KGTYKGS.........GIATVGGGKFSLM
ScMnn5     REVHSKFT.DTLSGKLNF......SIP..QREALFSGS........EGIVTIGGGKYSVL
CaMnn2     KDSHKRYVDEHIPKMLQRVKTFGSLAPSDKEWESYKGS........SGYIIVGGGRFTWL
CaMnn21    KKSHKYVV.ENLPE..........DAP....DGLYKKN.........GIVYVAGGSFNWL
CaMnn22    RQYHESVL.RNLPS..........KLP....KSVYTPNT.......YGIVTIGGNFYSWM
CaMnn23    KEKHSGVV.EDLPS..........VMP....GSVYNKGS.......KGVVIIGGGKFSWL
CaMnn24    KKSHTKFL.ETMME..........NWP....ENLFKFNKFNNFMKGDGIVYLGGGKYNQL
CaMnn26    RSAHDQVV.KQLKSLP........DPP....SQFISGH.........GIVVNGGGNMIGS
```

```
              250       260       270        280        290
OpoAbv2    ALLGIETLRKVGSTLPVEVILPSDDEY..EFEYCDQILP.ALNARCVEMPRVF.......
OpoMnn2A   SLLGIENLRATGSKLPVELIFPTEAEY..EEMLCEKVLP.DLNAKCVLLTERV.......
OpoMnn2B   ALLSIKTLRSVGSKLPVEILIPKLDEY..EVDLCTTIFP.ALNAKCIYMPKQL.......
ScMnn2     AFLIIKTLRNMGTTLPVEVLIPPGDEG..ETEFCNKILP.KYNSKCIYVSDIL.......
ScMnn5     AYTMIKKLRDTGTTLPIEVIIPPQDEG..EDDFCKNWLP.KFNGKCIYFSDIV.......
CaMnn2     SFLVIKQLRATGAKLPVEMFIATESDY..EKEFCEKVLP.KYNARCNVFDYKL.......
CaMnn21    TLLSIKSLRAVGCHLPIEVFIPKIEEY..ESDLCNRILP.ELDARCIYMKNQLMNPNKDN
CaMnn22    AYIQLLQLRKLGSNLPVEILIPSIEDYYKEAHFCDHVLP.QYNAKCILVPEKL.......
CaMnn23    AYLALVQLRNVGSKLPVEIVMPSRADYEKELEFCENTLP.EMQASCVVLPDVL.......
CaMnn24    VLLSIKILRENGSRLPVEVIIPYKNDY..DIQFCDRVLP.TLNGKCKLMTDYL.......
CaMnn26    ALTAIANMRERGSQLPVELILDTKQEY..DKQICEELLPKKLNGKCVIVEEQV.......
```

```
              300       310       320       330       340       350
OpoAbv2    GKTTLRKFDVNGYQFKAFALFASTFENAFFLDSDAYPVANPDPLFESDLYKEYQMITWPD
OpoMnn2A   PEFRKHKYTIRGYQYKILALLVSSFEQVLFLDSDNVPVANPDAIFVSEPFTSHGMVTWPD
OpoMnn2B   GEQISERFSFFGYQYKALALMLSSFENVLLLDADNTPLHAPDHLFETEPFTSTGMVIWPD
ScMnn2     PRETIEKFVFKGYQFKSLALIASSFENLLLLDADNFPIKPLDNIFNEEPYVSTGLVMWPD
ScMnn5     PSKPLSDLKLTHFQLKVFGLIISSFKRIIFLDADNYAVKNLDLAFNTTSFNDTGLILWPD
CaMnn2     ADDLKKRFDIGGYQYKMLALLSSKFENVLYLDSDNFPTRNVDYLFESDLYKENNLLLWPD
CaMnn21    SDSFANKFEFKGYQYKALAILLSSFENVLLLDSDNIPAHSPEELFENDPFKSYGLVVWPD
CaMnn22    GFNVAKHWKFSSYQFKALALCLSSFQHVLILDSDNVVLSKPEKVFDSPVYRDNGMVLWPD
CaMnn23    GEAVMKNRKFASYQFKALALVVTSFEHILLLDSDNMIVSNPDEIFESKLYHQYGMITWPD
CaMnn24    PQTFVDK..ISGFQLKNIALLISSFERILYLDADNIPIRNPDVLFTNAPFTTKHLVVWPD
CaMnn26    GKEVFD.IINEKFSRKIMGLLVSSFDHIIAMDADNLAIKNVDNLLFTEPYLSTKMILWPD
```

```
              360       370        380        390                 400
OpoAbv2    FWRRTTSPYFYQITGQEIGPKQ.VRH.LNDMFTDPKYYESEL...........NADPYHN
OpoMnn2A   FWRRVTHPTYYKVVDRELGNEQ.VRN.NIDDVTPNKYYARD............TGHAFSS
OpoMnn2B   YWKRSTSPAFYDIVNIEIDEGHRVSH....GFQEYGKYTTP............NSPPDQA
ScMnn2     FWRRTTHPLYYDIAGIAVDKKKRVRN.SRDDITPPAVYTKDL..............KDLSD
ScMnn5     FWRRVTPPAFYNIIGSSINIGKRVRF.VSDDISPVSRYDPFVSNSNDYTPKERQEHFLKH
CaMnn2     AWARTTNPKYYEIAGVPVKENK.LRY.........SKYDEKQAGGKDKL.KPLSEYTFKD
CaMnn21    YWKRATSPYYYNIADIDVSEKYLGSK............YNEVEGQYTDLS.VEKGSVELDK
CaMnn22    YWERTISPEWYDIIGKPVVGNKQVRTGRF....PVNIHNMLT...........SELEINE
CaMnn23    YWKRTISPLFYDVAEIEVNENKRVRYNRFPLYNAPNVRSNIY...........TDQEREE
CaMnn24    LWRRSTSPHYYTIAGIEVDPNFKVRN.SYVDGDERGKYTDSM.................Y
CaMnn26    LWVKLTSPLYYKIARIEPGEIV.DRFGIPNDAS....FAEYI...........TKDKQSE
```

```
                 410       420       430       440       450       460
OpoAbv2    IPFHDREGTIPDWTTEAGEMLINKTLHFQTLLLALYYNFDGPYGYYPLLSQGGAGEGDKE
OpoMnn2A   MPLHDRAGALPDPSSESGQIAVDKRTHLRALLLALYYNYYGPQQYYPLFSQGGAGEGDKE
OpoMnn2B   PPLHQLKGAIPDPSSESGQLMLSKKTHSKVMLLALYYNMYGPNHYYPLLSQGSDGEGDKE
ScMnn2     VPLSDLDGTIPDVSTESGQLMINKTKHLATALLSLFYNVNGPTWYYPIFSQKAAGEGDKE
ScMnn5     VPLHDLDGTMPDLSSESGQMVIDKIRHFNTLLLALYYNVYGPTWYYKMISQGTAGEGDKD
CaMnn2     SWYHDFEGTLPDPTSETGMFMVNKSSHLKTLLLCLYYNVFGPQYYYPLLTQGSAGEGDKE
CaMnn21    IPLHQRLGSIPDPTSESGQLLISKKTHLKPLLLALYYNLYGPSHYYPLFSQGSDGEGDKE
CaMnn22    TRFHDLEGALPDLSTESGQVMFNKKTHGKVMLMTLYYNIFGPEIYYKLFSLGALGEGDKD
CaMnn23    VPFHDLQGSIAELSTESGQLIINKHTHGKTILLALYYNFYGPNLFYKLFSLGEQGEGDKD
CaMnn24    YSYHDCKGSIPEASSETGQLLINKKIHFQTLILAMYYNYYGPDYYYPLFSQGAAGEGDKE
CaMnn26    VHYHDLDNLPSTISVETGQMVFSKREHLKSLLLALYYNINGKDFYIDLLYQGAYGEGDRE
```

```
                 470       480       490        500       510
OpoAbv2    TFVAAANYYGLKYYQVYKLPDRAYGWYN..HEQNYEHSSIVQYDPLTDYSNLQ.......
OpoMnn2A   TFVAAAQHFGLPFYHVRKAV.DVIGYWQLQPEEHYTGVGMIQYDPVVDYKLVAAYKDWFS
OpoMnn2B   TFIAAAHVLKKSFYQVKKII.KSIGRWA...NDEFTGSAMGQFNCHEDYKLYK.......
ScMnn2     TFIAAANFYGLSFYQVRTRT.GVEGYHD...EDGFHGVAMLQHDFVQDYGRYL.......
ScMnn5     TFVAAAHALNMPYYQVRTNF.EFDGFFY..QKDDYKGLALLQHDFEQDYKQYQ.......
CaMnn2     TFIAAAHVMKEPWYQCARQF.KWTGYVSK.VDNKFTSKALAHYDPV..............
CaMnn21    TFLAATVTLGKRYYQVAKFL.VSLGHFKV.PGGDFEGCGMGQFDPQQDLEYIKLREQYAK
CaMnn22    TFAAAALACGEKYYQVASSI.RTLGYFDTTPGGGFHGMAMAQKNPQLDYQLFQ.......
CaMnn23    TFVAAAVVTRQDYYQVKSFI.KTFGYAD..SDDKFQGVSMGQRNPLIDRKHYE..DHVLA
CaMnn24    TFIAAAHKLDLPYYQVGEFN.REFGPIND.NTRKHEFYGMGQYDPIIDYYMST.....IT
CaMnn26    TIVPALHVMNERYSLTNHKV.HILGYDA..PNGKYSETTLGQTDPRDGFEFYQDWRKFLT
```

```
                520                        530       540
OpoAbv2    .QVKQNIRKAIELEG.................EDFKYDYEKFFTQFFL......PSNSKV
OpoMnn2A   AREKEHERRQASFIGRLRD..........WWAGDVVYDPARTFTGWFN......KTNSRP
OpoMnn2B   ...................................KYE..........NSFEHVDEVPKL
ScMnn2     .NAMESIGNKYG.GTKSAD..........AIKFDKNYSLEKYTEEFFDNEDLNAKNHVDV
ScMnn5     .KAQQKVKANIEEFSKL................DPDYTLDNGFLKTLMVND..DGSDLDI
CaMnn2     .QAQDTTR...............................................QDVDI
CaMnn21    IPEKDKEK.........................QHKFK..........LQHQVLEKGPEI
CaMnn22    .KTNQNFK.........................DLHLDWDNEAKDQFS.....ANNKIPI
CaMnn23    LLEKDSFK.....SSSIAD..........QIEQMKKFE..........NEDFDQHNSIPL
CaMnn24    TTQKDTKK.KINYNSPLPE................KYAANDE.DDTCSNYDFHLFQSSSL
CaMnn26    SRKLDTRLNPFQSGGYTSDLMKQFHDYKRQIYQDKQYEDEAA......VHRMITYKLPSI

         550       560            570          580
OpoAbv2    LFYHVHDPKMDPFNI.....VNNRYTFDLD.GNRI..RNLGE..................
OpoMnn2A   MFVHSNFPKLDPVSL.....RKDDLLFVTNGGARKRVRMYSD..................
OpoMnn2B   LFLHSNFPKLDPWSL.....KVDEVIYDPETGKRE..RLYGT..................
ScMnn2     MFIHSNFPKFDPYDL.....SKSNFL.TTN.GKPA..RSYTA..................
ScMnn5     MFIHASFYKADPWTL.....YHENRFIGPN.GEQV..RGFRK..................
CaMnn2     IFMHLSYPKFYPNWL.....ADNHDLVYADTDEHI..RMYSSI.................
CaMnn21    LFVHANFPKLNPWKL.....KQEKKIFDAK.GNRV..RLYGP..................
CaMnn22    FTMHCNIKKINPAAY.....MKDEKIANMD.EKRMNVRFYSNLKFKLNDDDIYSPDNEKE
CaMnn23    FTVHCNYPKLDPKLY.....MSRDDLYDEK.EKKLKYRLYGNLK........YTKEVVKD
CaMnn24    FFLHANWPKYYIEKLFLYSYDEDRGPVTND.GDKR..RLYGN..................
CaMnn26    LFLHCNHPKIDPLKN.....SKEA...DAEFGVYSR.RNMGL..................

                        590       600       610                   620
OpoAbv2    .....D..FPEF..DFDLENFIWNVINHYVCEKKIN....FKHF..EN......ADWNSL
OpoMnn2A   ........QSGL..DFDYELRQWQFIKKHFCDKAID....LNYLRVAG......VTSLDY
OpoMnn2B   ......EYLEAL..GFDFERVQWENMKFYICDLNLH....PKLFSDRD......IDHDEL
ScMnn2     ........LKKV.KNYDIELENFKVLNEYVCVNKNP....FKYL..DDLLGQDKTEWKRV
ScMnn5     ........PHRY..GMDFELFLFNDMRGSFCTTPKSQVIKFKYF..TDKV..NTPEWDAM
CaMnn2     ........NKNV..GYDFDLRVMQFFTEGLCPNYYDSKT.GKPI..EN.....IPHIDYT
CaMnn21    ......GMIKRI..GYDFEWFQWEGMKYLLCIYDVN....LQIF..QD......VKKSDL
CaMnn22    KNKSEKTDNDPN..EIDFELSRWQIVSEILCDQKIT....FQFL..KD......ENMDEV
CaMnn23    GETGTEASTDKV..QIDFELQQWQHMQDILCLKKIY....FTHF..ID......NDMNEL
CaMnn24    ......ELKKEL.GGYDFELNIMKNLHWCFCEEPLIDLIGIPVV..GS......KTRTDV
CaMnn26    .....PDKVEKLLEGKDWELRFHTISQWVACEAISKSSLYWEKI..AG......KSQQQV

                630       640       650       660
OpoAbv2    CDRFIQDQLQFLAESSRRIFSDHNKSPPPDLVESPEKAEKVE..............TPDD
OpoMnn2A   CD.FIEQELEFL.ENSQHLVNIRNATT.................................
OpoMnn2B   CT.EIKEHLEFL.KSTTN..........................................
ScMnn2     CG.YITDRLAFL.ESTHD................KAIAGK....................
ScMnn5     CE.YLTNHVNYL.ESTHK................EAMGEKN...................
CaMnn2     ND.YMGNHLMYVKDEEQNNIDRCNKVFIPHLKWLKETAEIPT..................
CaMnn21    CD.EILEHLRFL.ESTQNITD.......................................
CaMnn22    CQ.FVKNTIAWLGKKT............................................
CaMnn23    CQ.FIENQVAWL.SKSQN..........................................
CaMnn24    CI.AIKNHIEFL.EIS............................................
CaMnn26    CE.SVGKYIEFLKKDTFD.....NEAT..KLTILNQLGEKSQPKQPEINNNNNNNNNDDD

                670
OpoAbv2    V......AVE
OpoMnn2A   ..........
OpoMnn2B   ..........
ScMnn2     ..........
ScMnn5     ..........
CaMnn2     .......VVS
CaMnn21    ..........
CaMnn22    ..........
CaMnn23    ..........
CaMnn24    ..........
CaMnn26    NGKNKQGAAS
```

Figure S4. Multiple alignment of amino acid sequences of Mnn2 homologs from *O. polymorpha* (Opo), *S. cerevisiae* (Sc), *Candida albicans* (Ca), generated using MAFFT and visualized in ESPript with BLOSUM62 matrix. Red background indicates strictly conserved residues; blue boxes highlight position with physicochemical similarity. OpoMnn2A, the polypeptide encoded by the ORF locating at 1396245-1398152 positions of the scaffold NW_017264699.1; OpoMnn2B, XP_018209282.1.

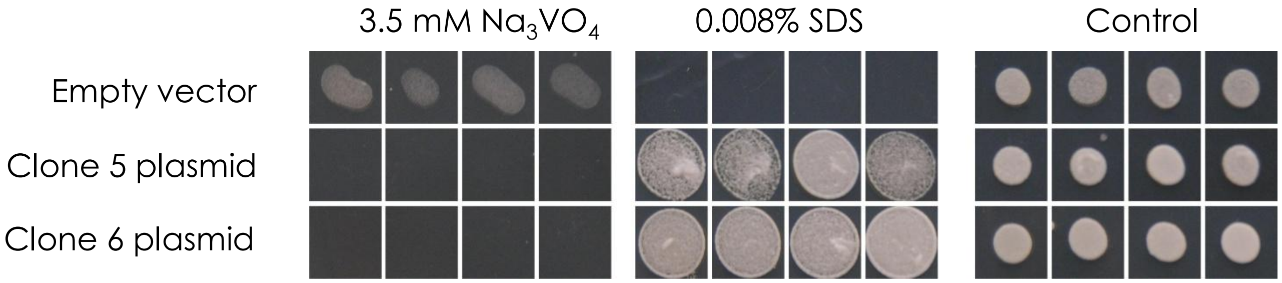

Figure S5. Complementation of AV35 mutant phenotypes by plasmids recovered from the genomic library. The AV35 strain was transformed with plasmids recovered from SDS-resistant transformants (clones 5 and 6; see also Figure S2), which were obtained by AV35 transformation with genomic library, and with the AMIpSL1 empty vector. Cells from colonies of subclones of the obtained transformants (4 transformants in each plasmid) were suspended in sterile water and spotted onto YPD plates supplemented with 3.5 mM Na3VO4 or 0.008% SDS using a microbiological replicator. The plates were incubated at 37°C for 2 days. The control plate did not contain the supplements and was photographed after 1 day incubation.

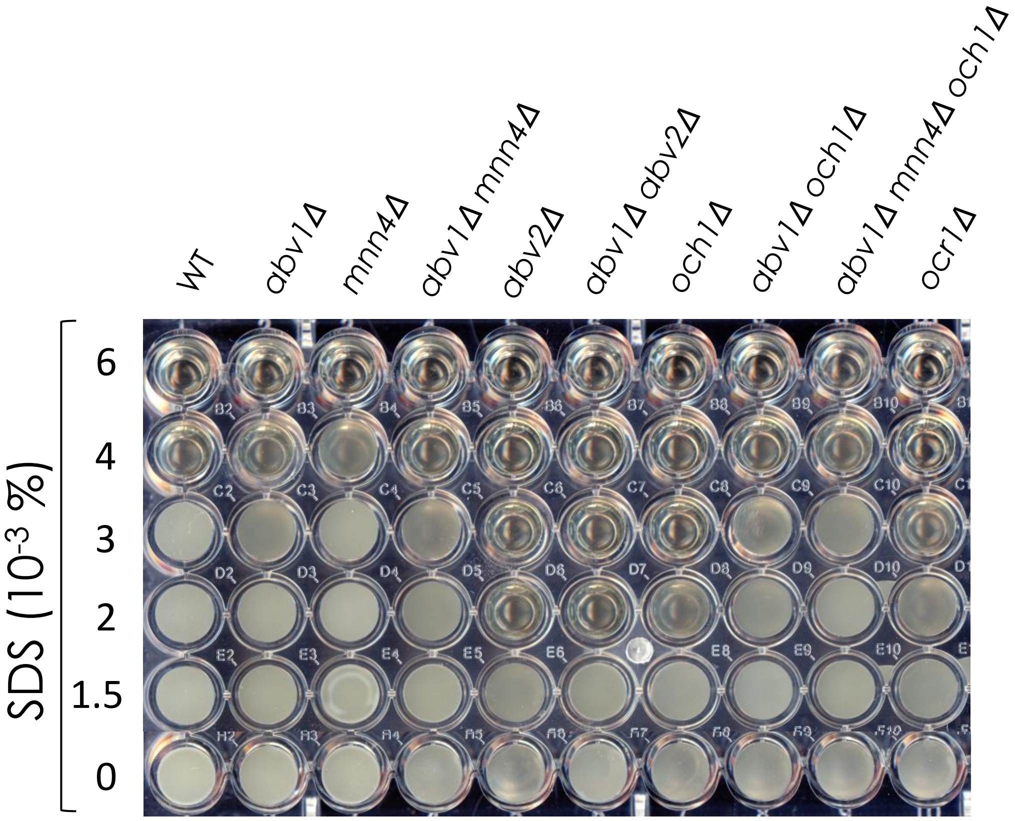

Figure S6. Growth of M257 (WT), M257-620M1 (*abv1Δ*), M257-MP9 (*mnn4Δ*), M257-620-MP9 (*abv1Δ mnn4Δ*), M257-759M1 (*abv2Δ*), M257-620-759M1 (*abv1Δ abv2Δ*), M257-MP6 (*och1Δ*), M257-620-MP6 (*abv1Δ och1Δ*), M257-620-MP9-MP6 (*abv1Δ mnn4Δ och1Δ*) and M257-1055 (*ocr1Δ*) strains in liquid YPD supplemented with SDS in different concentrations.

```
           1
KpPno1     MT...................LRSAIKA................................
KpMnn4A    MK..........................................................
OpoMnn4    MT....................SASNKR................................
ScMnn4     ML...................QRISSK.................................
ScMnn14    MM..........................................................
YlMpo1     MV..........................................................
KpMnn4C    MS.GNPFLFSPSNFDFSGLDHYRSTDKDHLALDVLDYDKNHFFSRNSPSLKSRIHFYRHK
OpoAbv1    MAQGDEELFIQ........EPPRAARKESHAS.........FILKAWLALVAPFTFV...
KpMnn4B    MF....................KETSKNLFGS............................

                        10        20        30              40
KpPno1     ..............RTSKGLIGAVIIASIIFFTTVTF......YDESKIVGIIR..VSDT
KpMnn4A    ......VSKRLIPRRSRLLIMMMLLVVYQLVVLVLGLES....VSEGKLASLLD..LGDW
OpoMnn4    ......YAVMMVPRRRRCVTLIAAICLLNLVIVSF........LGAGTQMDVLNDVTGLI
ScMnn4     LH.RRFLSGLLRVKHYPLRRILLPLILLQIIIITFIWSNSPQRNGLGRDADYLL......
ScMnn14    LSLRRFSMYVLRSLRLHFKKIIITLLTIQLLFITIFV........LGGRSSII.......
YlMpo1     LH.......PFRLLRTSLVSKLVVILITCLIFGSL.........................
KpMnn4C    LT.TRKQIGLFSGRLKLFVLALFVLITFSAIHIPIPF.......................
OpoAbv1    LN.KFIQLLPYHNNRKMSVFTIYIGLMLVFIVPTIFYMDESI..................
KpMnn4B    IN................TFNTVEYVMYMMLLLTAYFLNHL...................

            50          60        70        80        90        100
KpPno1     YTGHSAVSSTFN..ASSVVSDNKINGYGLPLIDTESNSRYEDPDDISIENELR.YRIAQS
KpMnn4A    DLANSSLSISD.....FIKLKLK............GQKTYHKFDEHVFA.....AMARIQ
OpoMnn4    RATSSSTDPDD.....FIKSFEDQ..............................LDSLDI
ScMnn4     .PNYNELDSDDDSWYSILTSSFK.............NDRKIQF.......AKTLYENL..
ScMnn14    ..........DGNWKSFMALFFK......PLAYTNRNNNHASFDLRSKDNVAKLYEKM..
YlMpo1     ......LNLTDK.LPDGVKSRVA...............................YMTDVG
KpMnn4C    ........SLD.....ILGSHVK...............................YLPLRE
OpoAbv1    ................FVMSKNK...............................Y.....
KpMnn4B    ......LHSLDN.INHLVESDVN...............................YQLLQR

              110       120       130       140       150       160
KpPno1     TKEEENMWKLDTTLTEASLKIPNIQSFELQPFKERLDNSLYNSKNIGNFYFYDPRLTFSV
KpMnn4A    SNENGKLADYESTSSKTDVTIQNVEL.....WKRLSEEE..........YTYEPRITLAV
OpoMnn4    SQNYGKYWKFSTTLNNAVVTFD......PSDYKTTRESR..........LLYDPRITMSV
ScMnn4     KFGTNPKWVNEYTLQNDLLSVK......MGPRKGSKLESVDELK....FYDFDPRLTWSV
ScMnn14    NFDTSGKWIDTYTLKNNLLTVK......MGPEKGQVLDSVDELR....YYDNDPRLVWSV
YlMpo1     LVSGGA..RSKAMAGNSSVRISHVPLT....KIKFAENE..........KEFNPKWAQKK
KpMnn4C    KVDPEEALH....LHGLDLSVAELPF.....FNDDMMSE..........FNYDPRLPTAL
OpoAbv1    EVDSEASFRKLQKSHTNDLAYV......ILPDEKHTEPHAPSK......SLFDQRVAVAA
KpMnn4B    VTNKVKLFDEEAVL..........PF.....AKNLNRRT..........ERFDPRLPVAA

              170              180       190                  200
KpPno1     YLKYIKDKLASGSTT.......NLTIPFNWAHFRDLSSL...........NPYLDIKQ..
KpMnn4A    YLSYIHQRTYDRYATSYA..PYNLRVPFSWADWIDLTAL...........NQYLDKTK..
OpoMnn4    YLSHIRRQLLEQGSGNAT..LAKIAVPFSWQDWVDLSVL...........NRYLELPE..
ScMnn4     VLNHLQNNDADQPE..........KLPFSWYDWTTFHEL...........NKLISIDK..
ScMnn14    LLDHLLESDSN.............EYAFSWYDWANFDST...........NKLIALRH..
YlMpo1     ALDS..................SSDYSFEWKDWVDLTEV...........TGLFRAIEVG
KpMnn4C    ILKLVLDHISVRNGTFDA....KFKVPFNWKLWVDLHSRLVPS.......NSWYNRFRLP
OpoAbv1    WLSNLKKHLMDNNGVLD....PDFQLPFSWAEWVDLESKLTLDKRHLHLWKSYHPEMQNR
KpMnn4B    YLRSLQDQYSELPQGTDLNDIPPLEVSFHWDDWLSLGIA...........STFWDAFD..

                210                          220       230
KpPno1     .EDKVACDYFYESS....................NKDKRKPTGNCIEFKDVR....DEHL
KpMnn4A    .....GCEAVFPR..........ESEATMKLNNITVVDWL.EGLCITDKSLQNSVNSTYA
OpoMnn4    .DQRPTCDDILYQ..........GPAYNPEHKFKNPNRNI.DRACVDNDRYL........
ScMnn4     ..TVLPCNFLFQSAFDKESLEAIETELGEPLFLYERPKYA.QKLWYKAARNQDRIKDSKE
ScMnn14    ..TNISCQFVCEGAFDKNVLEMVESEVQEPLFVTNRNKYD.ESLWYNRVR...KVVDSNS
YlMpo1     .....SCG....................................................
KpMnn4C    SGRFETCDEF......K........................RFFGITKNHFGTDLDNCVD
OpoAbv1    TFSDLTCADF................................RLLYADKDEV..LQVCSE
KpMnn4B    ..................................NYNKRQ.GENAISYEQLQ........
                                                       *
```

```
              240                  250                 260        270        280
KpPno1    IQY.........GISSKDHLPG......PFI.LKSLGIPMQHTAKRLESNLYLLTGAPVP
KpMnn4A   EEI...........NSRDILSP......NFHVFGYSDAKDNPQQKIFQSKSYINSKLPLP
OpoMnn4   ............GGTRRALLP.......GFN.FKDRTGKKSFRDKLIHAKSYLLSYAPVP
ScMnn4    LKK....HCSKLFTPDGHGSPKGLRFNTQFQ.IKELYDKVRPEVYQLQARNYILTTQSHP
ScMnn14   VQQAIHDHC.....MNNDAYSNGTPFELPFI.ISEISERLRPEVYDLQAKNHLLYSNFTP
YlMpo1    ..............KNKQCLS.......KLQVTGPPTQVEPQAFFNRVGKVFLDQTMPKP
KpMnn4C   IEY...........DTPEGYP.......KFKVLHAEDKALPYEARIIYGASYLYHEAQNP
OpoAbv1   LSH...........EERTQYP...WYPFSFKITDRTGSELAEAGRILFGASYMLTTAPPP
KpMnn4B   ............................AIL.VNDLEDFSPYTAHILHSNVEVYKYRTIP

                   290       300
KpPno1    LSLSFM...TKKGLYQVGVDQTGK....................................
KpMnn4A   KSLIFL...TDGGSYALTVDRTQ.....................................
OpoMnn4   TSIVFL...TENGSYEVNISGDV.....................................
ScMnn4    LSISII..ESDNSTYQVPLQTEK.....................................
ScMnn14   LSLTVL..DSDKDAYRINLKTTDS....................................
YlMpo1    KQLIYLTEKESRGKREEPVEIES..HVPVVREPELGDFSRSRDAKSIQNAKREATEEATG
KpMnn4C   KRLIFLGLGKSNESLILPVEAND.....................................
OpoAbv1   ARVHFIGAGLNKSSVVLLTERDAVTHMPI...............................
KpMnn4B   QKIVYM...SNKGYFELLVTEKEK....................................

                    310                 320                   330
KpPno1    LDPN......IARTELWEFYK........NGKENL..........QFNAQEELSH..LIE
KpMnn4A   .NKR......ILKSGLLSHFF........SKKKKEHN..LPQDQKTFTFDPVYEFNRLKS
OpoMnn4   ...N......MLDSGLFDEFV........ELQANE..........TVVADASLEHEMLVN
ScMnn4    .SKN......LVQSGLLQEYINDNINSTNKRKKNKQ.............DVEFNHNRLFQ
ScMnn14   SKSN......IVQTNLLQNYI........KRHRNE..........MVNGDLIFNHTSMFE
YlMpo1    ESANEGQSRDSARASARDIAF........SEADPVHEDSYDDDENGTILVPTAESEEELQ
KpMnn4C   .SSN......LMQFN..HEYA........................RSFNDQPFVSLEELVK
OpoAbv1   .SSNR..SRDIYSIG..AELVSNEARLRGVSVDRVHKDGVRIDQMVSELSTLFNYDTSVQ
KpMnn4B   ..........LSNEGLWSIFH........QKQGGLNE...............FSSLNLIE

           340                  350           360         370        380
KpPno1    TVPSSSNSSSG........EGYFTTE..LK.ENNF....ELPLSKNDFTFDDSEVESLIK
KpMnn4A   QVKPRPISS..........EPSIDSA..LK.ENDY....KLKLKESSFIFNYGRILSNYE
OpoMnn4   SVSPSKS............ELDFDSL..FTAEKNY....EFVIPPDRFNYSYDQIIENYE
ScMnn4    EFVNNDQVNS.....LYKLEIEETDK..FTFDKDL.....VYLSPSDFKFDASKKIEELE
ScMnn14   KFLHHGSTKK......RKLDVEALDK..TIYAGEY.....LELSPSDFQFNAKERIIELE
YlMpo1    EYADKDAAA..........KSYLRSG..WSRGQKY.....VELPRELFTWDIHEEIDKGL
KpMnn4C   KVLSTLNLNSDKVLPINELDVIKDTPRLMNHNNQG.....LSIDKSSFQWDLERELQLLE
OpoAbv1   TIYGED............ELVFDKSYFLKLDDQVKVMTSLEMTKQDFVYEHEEIMNAMK
KpMnn4B   EV.............DALDEIYDSKGLPAWDPPFP..EELDASDEDFKFNATEELAKVE

                  390                  400        410          420
KpPno1    ...GLSEQ.DLDLH.......TQRYKESLQYSFATREN..DVKKYFYEARMIINTVN...
KpMnn4A   ..ERLESLNDFEKS..........HYESLAYSSLLEAR..KLPKYFGEVILKNP......
OpoMnn4   ..KRIDEL...DEK.......QLRHLQTLKYSRSIPST..KLKKSFREVNINWPATYNGH
ScMnn4    EQKKLYPD.KFSAH.......NENYLNSLKNSVKTSPA..LQRKFFYEAGAVKQY.....
ScMnn14   ..TRLRSE.GLPSH.......DTHYLRSLKTSVNTSPA..LQQKYFAEASDITDA.....
YlMpo1    TKKSVDSD.DPSRE.......QVAHSQFLSQHWKHIK...KSGKHFSEAWVVGDT.....
KpMnn4C   ..HRTSQVNDVEGL.......DAGIYSTIQCEMRSMY...DFSKYFHESKVSGKY.....
OpoAbv1   ..QRIEKQLETKPDTVVDSLFDHLLLKNIEREMDLFEANGRHKPYLREAYVNN.......
KpMnn4B   ...QIKEP.KLE.........DIFYQEGLQHGIQTLPS..DASVYFPVNYVENDP.....
                                   *

            430       440       450       460       470       480
KpPno1    ..KEGGAHYDWRFFNGAMNHESSGFTEEERQLRKRSVLHRLLRNWLVFNYQQGSPTWLAH
KpMnn4A   ..QDGGIHYDYRFFSGLIDKTQINHFEDETE.RKKIIMHRLLRTWQYFTYHNNIINWISH
OpoMnn4   KVTENGGHYDFRFFNGFVTESKLNEYDDVNE.KRKIMLHRIIHTWLQFTYKEGIVSFLAH
ScMnn4    ..KGMGFHRDKRFFNV...DT...LINDKQE..YQARLNSMIRTFQKFTKANGIISWLSH
ScMnn14   ..TADGHHRDRRFFSI..GHN...LLNDPQE..FEARLNSLIRNFQKFVKANGLISWLSH
YlMpo1    ..KAAGVHYDWRFFSE.........LNTIDE..KRVILRKLVRAWLDFTSREGIITWLAH
KpMnn4C   ..LPSGEHYDWRFFNGFYL........SQQE..NLAVLHRLGRAWLRFSRAAGLHTWIAH
OpoAbv1   ..THLGAHFDWRFFNG.........IDHPQE.YRKAIIHRIGRAWLRFCYQSGFRTFVAY
KpMnn4B   ..GLQSHHLHFPFFSGMVL.........PRE..IHSSVHHMNKAFFLFARQHGYVVWFFY
            *                        **
```

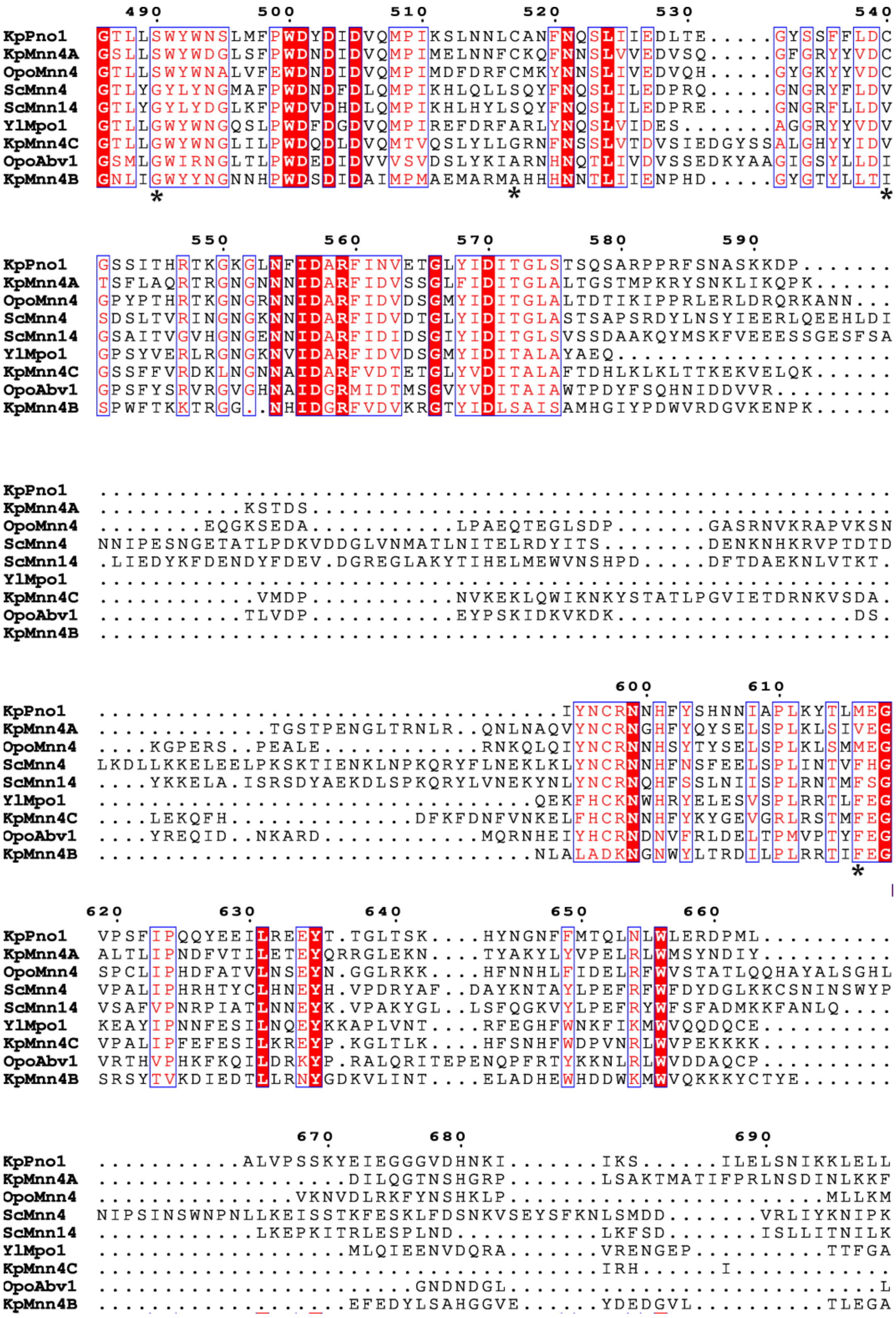
490 500 510 520 530 540
KpPno1 GTLLSWYWNSLMFPWDYDIDVQMPIKSLNNLCANFNQSLIIEDLTE.....GYSSFFLDC
KpMnn4A GSLLSWYWDGLSFPWDNDIDVQMPIMELNNFCKQFNNSLVVEDVSQ.....GFGRYYVDC
OpoMnn4 GTLLSWYWNALVFEWDNDIDVQMPIMDFDRFCMKYNNSLIVEDVQH.....GYGKYYVDC
ScMnn4 GTLYGYLYNGMAFPWDNDFDLQMPIKHLQLLSQYFNQSLILEDPRQ.....GNGRYFLDV
ScMnn14 GTLYGYLYDGLKFPWDVDHDLQMPIKHLHYLSQYFNQSLILEDPRE.....GNGRFLLDV
YlMpo1 GTLLGWYWNGQSLPWDFDGDVQMPIREFDRFARLYNQSLVIDES.......AGGRYYVDV
KpMnn4C GTLLGWYWNGLILPWDQDLDVQMTVQSLYLLGRNFNSSLVTDVSIEDGYSSALGHYYIDV
OpoAbv1 GSMLGWIRNGLTLPWDEDIDVVVSVDSLYKIARNHNQTLIVDVSSEDKYAAGIGSYLLDI
KpMnn4B GNLIGWYYNGNNHPWDSDIDAIMPMAEMARMAHHHNNTLIIENPHD.....GYGTYLLTI
550 560 570 580 590
KpPno1 GSSITHRTKGKGLNFIDARFINVETGLYIDITGLSTSQSARPPRFSNASKKDP.......
KpMnn4A TSFLAQRTRGNGNNNIDARFIDVSSGLFIDITGLALTGSTMPKRYSNKLIKQPK......
OpoMnn4 GPYPTHRTKGNGRNNIDARFIDVDSGMYIDITGLALTDTIKIPPRLERLDRQRKANN...
ScMnn4 SDSLTVRINGNGKNNIDARFIDVDTGLYIDITGLASTSAPSRDYLNSYIEERLQEEHLDI
ScMnn14 GSAITVGVHGNGENNIDARFIDIDSGIYIDITGLSVSSDAAKQYMSKFVEEESSGESFSA
YlMpo1 GPSYVERLRGNGKNVIDARFIDVDSGMYIDITALAYAEQ.....................
KpMnn4C GSSFFVRDKLNGNNAIDARFVDTETGLYVDITALAFTDHLKLKLTTKEKVELQK......
OpoAbv1 GPSFYSRVRGVGHNAIDGRMIDTMSGVYVDITAIAWTPDYFSQHNIDDVVR.........
KpMnn4B SPWFTKKTRGG..NHIDGRFVDVKRGTYIDLSAISAMHGIYPDWVRDGVKENPK......
KpPno1 ....
KpMnn4A ....KSTDS..
OpoMnn4 ........EQGKSEDA...........LPAEQTEGLSDP.......GASRNVKRAPVKSN
ScMnn4 NNIPESNGETATLPDKVDDGLVNMATLNITELRDYITS........DENKNHKRVPTDTD
ScMnn14 .LIEDYKFDENDYFDEV.DGREGLAKYTIHELMEWVNSHPD.....DFTDAEKNLVTKT.
YlMpo1 ....
KpMnn4C ............VMDP...........NVKEKLQWIKNKYSTATLPGVIETDRNKVSDA.
OpoAbv1 ...........TLVDP...........EYPSKIDKVKDK.................DS.
KpMnn4B ....
600 610
KpPno1 ..................................IYNCRNNHFYSHNNIAPLKYTLMEG
KpMnn4A .............TGSTPENGLTRNLR..QNLNAQVYNCRNGHFYQYSELSPLKLSIVEG
OpoMnn4 ....KGPERS..PEALE...........RNKQLQIYNCRNNHSYTYSELSPLKLSMMEG
ScMnn4 LKDLLKKELEELPKSKTIENKLNPKQRYFLNEKLKLYNCRNNHFNSFEELSPLINTVFHG
ScMnn14 ....YKKELA.ISRSDYAEKDLSPKQRYLVNEKYNLYNCRNQHFSSLNIISPLRNTMFSG
YlMpo1 ..............................QEKFHCKNWHRYELESVSPLRRTLFEG
KpMnn4C ....LEKQFH.............DFKFDNFVNKELFHCRNNHFYKYGEVGRLRSTMFEG
OpoAbv1 ....YREQID..NKARD...........MQRNHEIYHCRNDNVFRLDELTPMVPTYFEG
KpMnn4B ................................NLALADKNGNWYLTRDILPLRRTIFEG
620 630 640 650 660
KpPno1 VPSFIPQQYEEILREEYT.TGLTSK....HYNGNFFMTQLNLWLERDPML..........
KpMnn4A ALTLIPNDFVTILETEYQRRGLEKN....TYAKYLYVPELRLWMSYNDIY..........
OpoMnn4 SPCLIPHDFATVLNSEYN.GGLRKK....HFNNHLFIDELRFWVSTATLQQHAYALSGHL
ScMnn4 VPALIPHRHTYCLHNEYH.VPDRYAF..DAYKNTAYLPEFRFWFDYDGLKKCSNINSWYP
ScMnn14 VSAFVPNRPIATLNNEYK.VPAKYGL..LSFQGKVYLPEFRYWFSFADMKKFANLQ....
YlMpo1 KEAYIPNNFESILNQEYKKAPLVNT....RFEGHFWNKFIKMWVQQDQCE..........
KpMnn4C VPALIPFEFESILKREYP.KGLTLK....HFSNHFWDPVNRLWVPEKKKK..........
OpoAbv1 VRTHVPHKFKQILDRKYP.RALQRITEPENQPFRTYKKNLRLWVDDAQCP..........
KpMnn4B SRSYTVKDIEDTLLRNYGDKVLINT....ELADHEWHDDWKMWVQKKKYCTYE.......
670 680 690
KpPno1 ...........ALVPSSKYEIEGGGVDHNKI.......IKS......ILELSNIKKLELL
KpMnn4A ...................DILQGTNSHGRP.......LSAKTMATIFPRLNSDINLKKF
OpoMnn4 ...............VKNVDLRKFYNSHKLP.......................MLLKM
ScMnn4 NIPSINSWNPNLLKEISSTKFESKLFDSNKVSEYSFKNLSMDD.......VRLIYKNIPK
ScMnn14 ...........LKEPKITRLESPLND...........LKFSD.......ISLLITNILK
YlMpo1 ...................MLQIEENVDQRA.......VRENGEP..........TTFGA
KpMnn4C ......................................IRH......I...........
OpoAbv1 ....GNDNDGL..........................L
KpMnn4B ...................EFEDYLSAHGGVE......YDEDGVL..........TLEGA

```
           700       710       720       730       740       750
KpPno1     DDNPDILEEVIRTYELTSIHHKEMQYLSSVKPDGDRSMQSNDITSSYQEFLASL.KKFQP
KpMnn4A    LRNDHTFKNIYSTFNVTRVHEEELKHLIVNYDQNKRK......SAEYRQFLENL.RFMNP
OpoMnn4    LGTDELLKEFYLTHKTTKMHLEEFSLM..NQLSDPQK......KERYYSIMEEYKRTHDP
ScMnn4     AGFIEVFTNLYNSFNVTAYRQKELEIQYCQNLTFIEK......KKLLHQLRINVAPKLSS
ScMnn14    CGFHSVFASLFNSFDSTVYRLKELEIQYDPSLSEEEK......SSLLKTLRRGMSKKIKS
YlMpo1     CYRPEYLKRYHETHKMSKAHEGEMEAIRQK.............ADVWEWIREEF......
KpMnn4C    ........EFSLTKEVTESHKKELAQIHGNET.........GITSDF.........AYSP
OpoAbv1    CRDEDVLEEYAHTKEYTARHLELLKHIYDNALSIEDE....TVPMRYDQFIVRYAQLLNA
KpMnn4B    CGFEEVRQDWIITRESVNLHMKEWEAIRQNES...........TTEYTA......KDLPR

            760
KpPno1     LRKDLFQF....................................................
KpMnn4A    IRKDLVTYESRLKALDGYNEV.......................................
OpoMnn4    MRKSLHGYISEVVALGGYHPQ.......................................
ScMnn4     PAKDPFLFGYEKAMWKDLSKSMNQTTLDQVTKIVHEEYVGKIIDLSESLKYRNFSLFNIT
ScMnn14    PEKDPIIYIYERKLWENVEKLLNASNIYNIASQVEKEKGKEFVERSQQVYERNFDGFRLP
YlMpo1     ............................................................
KpMnn4C    FRIDPWLSRYRKKMT.............................................
OpoAbv1    ............................................................
KpMnn4B    YRPDSF....KNLLDGVSNHGNG...NVGKI.............................

KpPno1     ............................................................
KpMnn4A    ............................................................
OpoMnn4    ............................................................
ScMnn4     FDETGTTLDDNTEDYTPANTVEVNPVDFKSNLNFSSNSFLDLNSYGLDLFAPTLSDVNRK
ScMnn14    DGGNSKTVN...............................DLNSKGLNLFG....DNKKT
YlMpo1     ............................................................
KpMnn4C    ............................................................
OpoAbv1    ............................................................
KpMnn4B    ............................................................

                                      770
KpPno1     .......................ERIDLSKH.............................
KpMnn4A    .......................EELEKKQENREKERKEKKEKEEKEKKEKEEKEKKEKE
OpoMnn4    .......................EEPARSALSETSEELDRVVITGKSQSNAGARRIQK..
ScMnn4     GIQMFDKDPIIVYEDYAYAKLLEERKRREKKKKEEEEKKKKEEEEKKKKEEEEKKKKEEE
ScMnn14    SNNIFGSD....................................................
YlMpo1     ............................................................
KpMnn4C    ............................................................
OpoAbv1    ............................................................
KpMnn4B    .......................EHVKLE...............................

KpPno1     ............................................................
KpMnn4A    EKEKKEKEEKERKEKEEKEEYEEDDNEGEQPTEQKSQQE.....................
OpoMnn4    ............................................................
ScMnn4     EKKKKEEEEKKKKEEEEKKKQEEEEKKKKEEEEKKKQEEGEKMKNEDEENKKNEDEEKKK
ScMnn14    ............................................................
YlMpo1     ............................................................
KpMnn4C    ............................................................
OpoAbv1    ............................................................
KpMnn4B    ............................................................

KpPno1     ...........................................RKQ
KpMnn4A    ...........................................AKE
OpoMnn4    ...........................................SPT
ScMnn4     NEEEEKKKQEEKNKKNEDEEKKKQEEEEKKKNEEEEKKKQEEGHSN
ScMnn14    ...........................................QKY
YlMpo1     .............................................E
KpMnn4C    ...........................................RSQ
OpoAbv1    ...........................................RLE
KpMnn4B    ...........................................HND
```

Figure S7. Multiple alignment of amino acid sequences of Mnn4 homologs from *O. polymorpha* (Opo), *K. phaffii* (Kp), *S. cerevisiae* (Sc), *Y. lipolytica* (Yl), generated using MAFFT and visualized in ESPript with BLOSUM62 matrix. Red background indicates strictly conserved residues; blue boxes highlight position with physicochemical similarity. Asterisks mark positions distinguishing Mnn4 from Abv1 protein groups.

Table S1. Oligonucleotides

Name	5'-3' sequence
ABV2U	CGAGTTGGAAGGAGAAGAC
ABV2L	GCTGCCTGGTTTGGATTG
OpoMNN4AU1	CACATCAAACCCGCTCGCTG
OpoMNN4L1	CCCGTTTGCCACTGTGTGCTG
OpoMNN4AL1	GTGCTCTACAATCTCCTGTCC
OpoMNN4U1	CTCACAAGACCACCAAGATGC
OpoOCH1U1	GAAGACAAGATCGAGTGGGA
OpoOCH1L1	GTTCTTCTGGATCTCGCTTG
OpaOCR1AL	CGGTGGTGAACGCGGTGGTG
OpaOCR1AU	GGGGCACAGATGACAAAATG
cbsCTS1_L2	CTGTTTCCTCCTGTTTCTCCTG
hpcts5'	TTGGATCCACAGCCCAGGCTTTTG
